# Anxiolysis in the Surgical Management of a Compound Odontoma in a Pediatric Patient

**DOI:** 10.1155/2019/1385150

**Published:** 2019-04-02

**Authors:** Pier Paolo Poli, Luca Creminelli, Emma Grecchi, Silvia Pieriboni, Gregorio Menozzi, Carlo Maiorana

**Affiliations:** ^1^Department of Biomedical, Surgical and Dental Sciences, University of Milan, Via Commenda 10, 20122 Milan, Italy; ^2^Oral Surgery Department, Maxillofacial Surgery and Odontostomatology Unit, Fondazione IRCCS Cà Granda Maggiore Policlinico Hospital, University of Milan, Via Commenda 10, 20122 Milan, Italy; ^3^Oral Surgery and Implantology Departments, Maxillofacial Surgery and Odontostomatology Unit, Fondazione IRCCS Cà Granda Maggiore Policlinico Hospital, University of Milan, Via Commenda 10, 20122 Milan, Italy

## Abstract

Among odontogenic tumors, odontoma is the most frequent. The common treatment contemplates a conservative approach. While this procedure is generally accepted and tolerated, some difficulties may be encountered in the case of pediatric patients. Indeed, negative feelings of tension, apprehension, nervousness, and fear are likely to occur. The present report is aimed at discussing the management of a compound odontoma in a pediatric patient under anxiolysis with diazepam on an outpatient basis. The surgery was carried out without complications, and the discharge was completed safely. Oral premedication with diazepam should be considered to avoid more invasive sedation procedures in anxious pediatric patients.

## 1. Introduction

Odontomas are benign calcifying odontogenic tumors of the oral cavity. Generally, odontomas are considered to be hamartomatous developmental malformations of dental tissues rather than true neoplasms, as both epithelial and ectomesenchymal components have morphologically normal cells with defective structural arrangement [1]. Two basic types of odontoma can be identified: complex and compound. The complex type is characterized by unorganized dental tissue with dysplastic dentin covered by enamel, resulting in haphazard amorphous calcifications. The compound odontoma forms multiple disordered and irregular tooth-like elements [2]. Complex odontomas most frequently occur in the posterior molar regions, whereas compound odontomas tend to develop in the anterior maxilla [3]. The incidence of odontogenic tumors is low, ranging from 0.002% to 0.1%, with the odontoma representing the most common entity, accounting for 20% to 67% of all odontogenic neoplasms [4]. The multifactorial etiology of odontomas is still debated. Local trauma in the primary dentition period, infection growth pressure, hereditary anomalies, family history, and developmental influences have been suggested as possible causes [5]. Most lesions are detected on routine radiographs as a consequence of their slow and painless growth. Eruption disturbances including impaction, delayed eruption, or retention of primary or permanent teeth are common clinical findings [6]. While these lesions can be diagnosed at any age, it is worthy of note that odontomas are considered to be the most common types of odontogenic tumors in the pediatric population [3]. Pediatric dentistry often involves patients with high levels of anxiety and fear. Currently available evidence suggests that approximately 6% [7] to 40% [8] of children exhibit anxiety with respect to dental treatment. Furthermore, anxiety is exacerbated by invasive and long-lasting procedures including surgical interventions [9]. Anxious patients are likely to express subjective feelings of tension, apprehension, nervousness, and fear that might compromise dental treatment. For such reasons, anxiolysis has been used to facilitate dental care for anxious and uncooperative pediatric dental patients with successful results [10]. In view of the above, this report is aimed at presenting a case of compound odontoma surgically managed in association with anxiolysis protocol in a pediatric patient on an outpatient basis.

## 2. Case Report

A 7-year-old healthy Caucasian boy was referred by the orthodontist to investigate the edentulous space between the first and second primary upper left molars, together with an unusual swelling in the same region. After an interview with the parents, a noncontributory medical history was confirmed.

**Figure 1:**
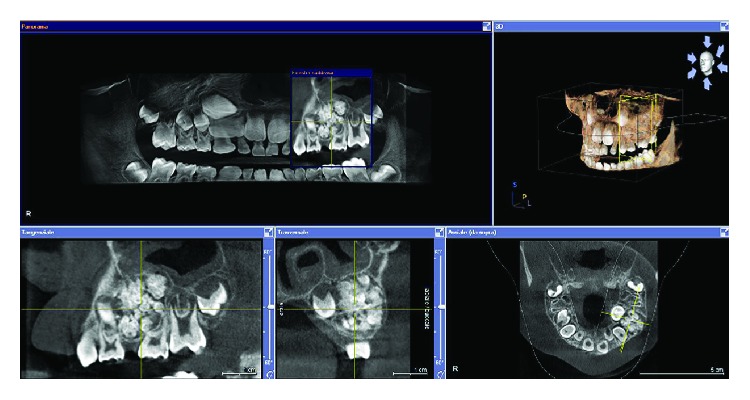
Cone-beam computed tomography showing radiopaque calcified structures with irregular margins characterized by a tooth-like configuration enclosed in radiolucent peripheral borders. The entire lesion measures approximately 12 × 15 × 19 mm.

The intraoral clinical examination revealed mixed dentition with no decayed teeth. A 6 mm edentulous space between the two primary upper left molars was observed. In addition, expansion of the cortical bone was present on the vestibular aspect of the right hemi-maxilla. The bony hard swelling was firm and asymptomatic. The overlying mucosa was normal and nontender on palpation. Second-level radiological investigation was performed by means of the cone-beam computed tomography (CBCT) scan, which confirmed the presence of an irregular radiopaque mass located by the roots of the primary molars (Figure 1).

In more detail, the well-corticated lesion was characterized by multiple radiopaque structures encapsulated within a radiolucent cavity. Moreover, the lesion progressed in a vestibular direction so that the permanent second premolar germ resulted dislocated palatally. As a matter of fact, the germ could be palpated on the palatal aspect of the edentulous ridge. The clinical and radiographic presentations led to an initial diagnostic hypothesis of compound odontoma. The suggested treatment plan consisted of surgical removal of the lesion under oral sedation of the patient on an outpatient basis. After discussing the aforesaid surgical procedure, an informed consent signed by the parents was obtained.

Before the surgery, the following vital parameters were recorded: arterial blood pressure (systolic/diastolic pressure ratio: 85/50 mmHg), peripheral capillary oxygen saturation (SpO2: 99%), and heart rate (84 bpm). The weight and height of the patient were also registered (26 kg and 126 cm, respectively). At this point, the anxiolysis protocol used in the department in the case of pediatric patients was adopted (Table 1).

In this specific case, premedication consisted of oral administration of 15 drops of diazepam [11] 3 mg/ml (Valium®, Roche Pharmaceuticals, Monza, Italy) and topical application of 15% lidocaine spray (OGNA Pharmaceuticals, Muggiò, Italy). A fingertip pulse oximeter was used during the entire procedure to monitor the oxygen saturation of the patient.

**Figure 2:**
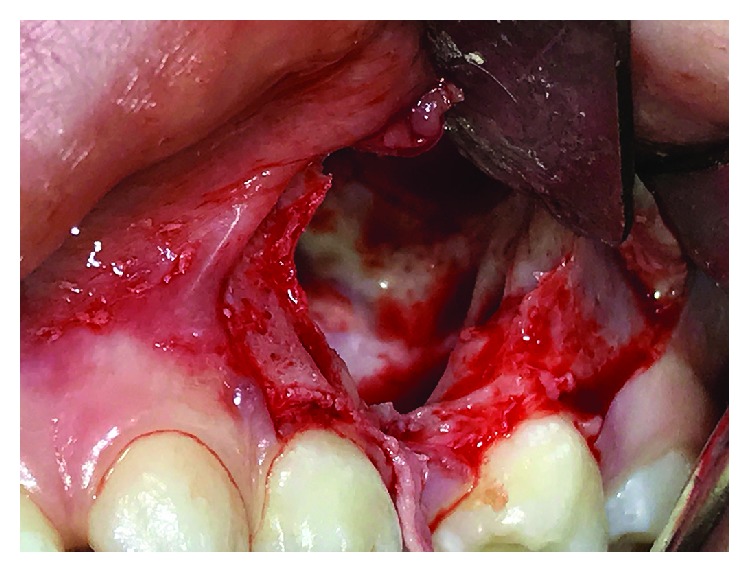
Intraoral clinical photograph of the surgical access showing the residual bone cavity after removal of the odontoma, with preservation of the adjacent maxillary sinus walls.

Local anesthesia was induced with infiltrations of mepivacaine hydrochloride 3% (Optocain® 30 mg/ml, Molteni Dental Srl, Milan, Italy). Crestal incision with vertical releasing incisions located at the mesial and distal aspects of the first and second primary molars, respectively, was performed to raise a trapezoidal mucoperiosteal flap. The odontoma was accessed by removing the thin overlying cortical layer with a round bur mounted on a straight surgical handpiece under copious irrigation of sterile saline (Figure 2).

**Figure 3:**
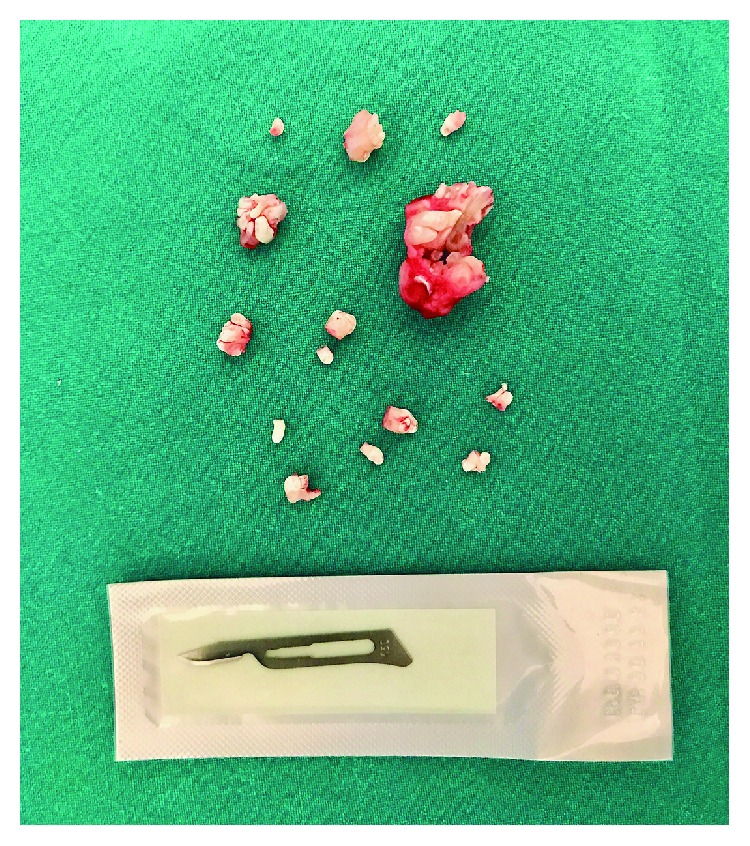
Clinical view of the compound odontoma surgically removed in multiple mineralized pieces resembling tooth structures.

The denticles were subsequently exposed and removed in a total of 14 pieces (Figure 3).

All harvested samples were sent for histopathological analysis. The surgical site was debrided and irrigated to remove any remnants, and the integrity of the maxillary sinus walls was checked. First intention sealing of the surgical wound was finally achieved with a 4/0 resorbable suture (Polysorb™, Covidien, Dublin, Ireland).

The time elapsed between the premedication and the end of the surgical procedure was 90 minutes. The vital parameters recorded immediately after the surgery were as follows: arterial blood pressure (systolic/diastolic pressure ratio: 80/50 mmHg), peripheral capillary oxygen saturation (SpO2: 100%), and heart rate (82 bpm).

The patient was prescribed antibiotic therapy consisting of 7.5 ml amoxicillin clavulanate pediatric suspension (Augmentin® pediatric suspension, GlaxoSmithKline, Verona, Italy) 3 times daily for 6 days and analgesics according to the patient needs. The rationale for antibiotic administration was to reduce the risk of infection of the blood clot during the immediate postoperative period in view of the considerable size of the lesion.

**Figure 4:**
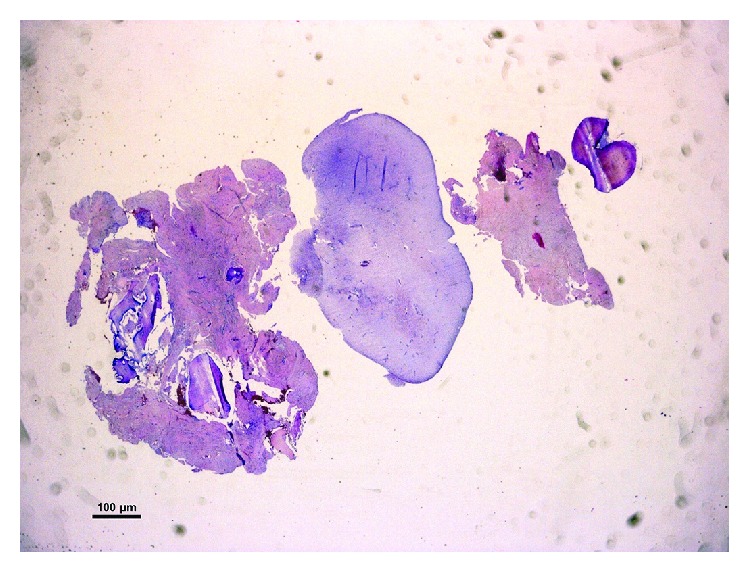
Low-power magnification of a resected specimen showing dental tissue, dentin, cement, and nonmineralized oral tissue composed of connective tissue typical of dental pulp, partially surrounded by a connective fibrous tissue capsule (hematoxylin and eosin staining; original magnification: ×10).

Postoperative care instructions included soft warm diet for 48 hours, 0.2% chlorhexidine mouthwash 2 times daily up to suture removal, topical application of ice, and refrainment from mechanical plaque removal of the primary molars for 7 days. The patient was discharged in stable conditions 90 minutes after the end of the surgical procedure. The healing proceeded uneventfully, and sutures were removed after 7 days. The histopathological report provided by the Department of Anatomic Pathology confirmed the diagnosis of compound odontoma (Figure 4).

## 3. Discussion

The present report described the surgical management of a compound odontoma detected in a pediatric patient with in-office sedation. Heterogeneity can be found among data reported from the literature with respect to the epidemiology of the disease. The present case is consistent with other studies reporting that compound odontoma is more frequently diagnosed in male subjects [6, 12] during the first two decades of life [13] in the premaxillary region [14]. Also, the clinical and radiological characteristics of the odontoma diagnosed in the present case resemble those commonly encountered for this type of lesion. Clinically, a firm bony swelling of the oral vestibule covered by normal mucosa with no signs of inflammation can be appreciated [15]. Although they are commonly asymptomatic, clinical manifestations may occur, such as retention of deciduous teeth, noneruption of permanent teeth, and tooth displacement [13]. These clinical findings, if recognized, might explain the early diagnosis achieved in the present report. Dentition anomalies, and in particular the retention of the permanent second premolar, have led to a radiological second-level investigation which confirmed the suspect of compound odontoma. As a matter of fact, the CBCT exams showed multiple radiopaque tooth-like structures of various sizes surrounded by a radio-lucid halo, compatible with compound odontoma at an advanced stage of development [13]. It is noteworthy that the mean age of patients at diagnosis of odontoma is normally between 20 and 30 years [4, 16, 17], with a mean age reported for compound odontoma of 20.5 years [17]. Conservative surgical enucleation and curettage were performed as the treatment of choice [3]. As mentioned above, extended dental treatments such as oral surgery procedures may cause deterioration of the pediatric patient behavior associated with dental fear and dental anxiety [9]. In particular, dental anxiety indicates an excessive and unreasonable negative emotional state that might jeopardize the outcome of the procedure [18]. In such cases, anxiolysis, better known as *minimal sedation*, might be advocated to induce a state of a minimally depressed level of consciousness with ventilatory and cardiovascular functions unaffected [19]. In the present report, this effect has been positively exploited to minimize the psychological trauma and to control behavior and movements of the pediatric patient. Among benzodiazepines, diazepam has been used for many years successfully. It has been claimed that diazepam represents the safer drug for anxiety management in dentistry if compared to midazolam due to its pharmacological profile [20]. Other authors observed no differences between the behaviors of children premedicated with oral diazepam or oral midazolam [21]. Conversely, emerging evidence provided better results for oral midazolam with respect to oral diazepam when the behavioral parameters including sleep, crying, movement, and overall behavior were compared [22]. In the present case, the immediate postoperative period was uneventful, and the patient was discharged safely 90 minutes postsurgery. It must be noted however that postdischarge excessive somnolence, nausea, and emesis have been frequently reported after oral sedation in pediatric dental patients [23]. Anxiolysis with oral administration of diazepam induced positive psychological responses to treatment and safe discharge. This procedure should therefore be considered in the case of potentially prolonged surgical interventions in pediatric dental patients to avoid more invasive sedation procedures.

## Figures and Tables

**Table 1:** Anxiolysis protocol adopted in the case of pediatric patients.

N_2_O/O_2_^a^
OR midazolam 0.50-0.75 mg/kg oral administration (maximum: 20 mg) or 0.2 mg/kg intravenous medication (maximum: 10 mg)^b^ ± N_2_O/O_2_^∗^
OR diazepam 0.2-0.5 mg/kg oral administration (maximum: 10 mg)^b^ ± N_2_O/O_2_^∗^
OR hydroxyzine oral solution 0.5 mg/kg ± N_2_O/O_2_^a^

^a^Medical nitrous oxide/oxygen mixture. The percentage of N_2_O is titrated for each patient during every procedure; ^b^the drug used as benzodiazepines antagonist is flumazenil 0.01 mg/kg (maximum: 0.2 mg) followed if necessary by additional 0.01 mg/kg every minute up to five times (maximum tolerated cumulative dose: 1 mg).
